# Regulation of Constitutive GPR3 Signaling and Surface Localization by GRK2 and β-arrestin-2 Overexpression in HEK293 Cells

**DOI:** 10.1371/journal.pone.0065365

**Published:** 2013-06-27

**Authors:** Katie M. Lowther, Tracy F. Uliasz, Konrad R. Götz, Viacheslav O. Nikolaev, Lisa M. Mehlmann

**Affiliations:** 1 Department of Cell Biology, University of Connecticut Health Center, Farmington, Connecticut, United States of America; 2 Department of Cardiology and Pneumology, Georg August University Medical Center, Heart Research Center Göttingen, European Heart Research Insitute Göttingen, Göttingen, Lower Saxony, Germany; Loyola University Chicago, Stritch School of Medicine, United States of America

## Abstract

G protein-coupled receptor 3 (GPR3) is a constitutively active receptor that maintains high 3′-5′-cyclic adenosine monophosphate (cAMP) levels required for meiotic arrest in oocytes and CNS function. Ligand-activated G protein-coupled receptors (GPCRs) signal at the cell surface and are silenced by phosphorylation and β-arrestin recruitment upon endocytosis. Some GPCRs can also signal from endosomes following internalization. Little is known about the localization, signaling, and regulation of constitutively active GPCRs. We demonstrate herein that exogenously-expressed GPR3 localizes to the cell membrane and undergoes internalization in HEK293 cells. Inhibition of endocytosis increased cell surface-localized GPR3 and cAMP levels while overexpression of GPCR-Kinase 2 (GRK2) and β-arrestin-2 decreased cell surface-localized GPR3 and cAMP levels. GRK2 by itself is sufficient to decrease cAMP production but both GRK2 and β-arrestin-2 are required to decrease cell surface GPR3. GRK2 regulates GPR3 independently of its kinase activity since a kinase inactive GRK2-K220R mutant significantly decreased cAMP levels. However, GRK2-K220R and β-arrestin-2 do not diminish cell surface GPR3, suggesting that phosphorylation is required to induce GPR3 internalization. To understand which residues are targeted for desensitization, we mutated potential phosphorylation sites in the third intracellular loop and C-terminus and examined the effect on cAMP and receptor surface localization. Mutation of residues in the third intracellular loop dramatically increased cAMP levels whereas mutation of residues in the C-terminus produced cAMP levels comparable to GPR3 wild type. Interestingly, both mutations significantly reduced cell surface expression of GPR3. These results demonstrate that GPR3 signals at the plasma membrane and can be silenced by GRK2/β-arrestin overexpression. These results also strongly implicate the serine and/or threonine residues in the third intracellular loop in the regulation of GPR3 activity.

## Introduction

G protein-coupled receptors (GPCRs) represent the largest family of integral membrane proteins and regulate a wide variety of physiological processes. GPCRs typically bind to an extracellular agonist which causes the receptor to adopt an active conformation. However, some receptors exhibit constitutive activity in the absence of a ligand. The level of constitutive activity varies among receptors and also seems to depend on the cell type [1]. Constitutive activity can be a property of the receptor itself or the result of chronic stimulation by a ligand, as in the case of the dog adenosine A2a receptor [2].

GPR3, GPR6, and GPR12 constitute a family of constitutively active G_s_-coupled GPCRs [3]. The magnitude of constitutive activity of these receptors is reported to be the highest among all GPCRs and is similar in amplitude to a ligand-stimulated GPCR [1], [4]. GPR3 is classified as an orphan receptor and it is thought to mediate sustained cAMP production in the absence of a ligand [4]–[6], although a membrane-bound ligand or activating GPCR-interacting protein cannot be ruled out. In the mouse, GPR3 is expressed highly in the brain, with lower amounts in the ovary, testis and eye [4]. GPR3 is essential for maintaining prophase I meiotic arrest in mouse and pig oocytes [7]–[10] and may play a role in meiotic arrest in human and *Xenopus* oocytes [11]–[13]. GPR3 has also been found to be important for several neurological processes including neurite outgrowth, postnatal cerebellar development [14], [15], emotional-like responses, Alzheimer’s disease, early phases of cocaine reinforcement, and neuropathic pain therapy [16]–[19]. Although the constitutive activity of GPR3/6 and 12 has long been recognized, little is known about the molecular details by which the signaling activity and subcellular localization of these receptors are controlled. Understanding GPR3 regulation may not only be important for understanding other constitutively active receptors, but may lead to therapies for reproductive and neurological disorders.

An important mechanism that regulates GPCR signaling is desensitization. This process involves the G protein-coupled receptor kinases (GRKs) and the β-arrestins [20], [21]. GRKs selectively phosphorylate active GPCRs at serine and threonine residues within the C-terminus and third intracellular loop. This leads to the recruitment of arrestin, which prevents subsequent interactions with the receptor and G proteins, thereby terminating G protein-mediated signaling [22], [23]. β-arrestin binding can also promote internalization of the receptor through a clathrin-dependent pathway. Following internalization, the receptor is either dephosphorylated and recycled back to the membrane or it is targeted to lysosomes for degradation. Although it is assumed that receptor internalization terminates GPCR signaling, there are recent reports of cAMP signaling by internalized GPCRs. The thyroid-stimulating hormone (TSH) and parathyroid hormone (PTH) receptors continue to signal following internalization where they remain associated with G proteins and adenylate cyclase at endosomal compartments. Signaling from internalized receptors is associated with a prolonged cAMP response following hormone treatment, whereas signaling at the cell surface is typically more transient [24]–[27]. The D_1_ dopamine receptor is an example of another GPCR that produces cAMP following internalization to support acute dopaminergic signaling [28]. In addition to G_s_-dependent signaling, there is evidence that G_i_-dependent signaling stimulated by the S1P receptor may occur internally [29]. Intracellular signaling seems to contradict the well-established process of desensitization; therefore, further studies are needed to reconcile these two concepts. It is not known if intracellular signaling to cAMP is a general feature of G_s_/G_i_-coupled receptors or if it is a characteristic of only a few receptors and/or only occurs in certain cell types.

GPR3 behaves like agonist-occupied receptors in that it uses traditional GPCR pathways to transmit signals and is internalized by an endocytic pathway [5], [30], [31]. However, it is unknown if the activity and localization of GPR3 and other constitutively active GPCRs are regulated by GRKs and arrestins. Since the constitutive activity of GPR3 is required to maintain high cAMP levels for meiotic arrest, it could signal following internalization in order to prolong cAMP signaling. Our previous findings show that GPR3 is internalized in the oocyte and inhibition of endocytosis increases cAMP and delays oocyte maturation [31]. This finding is consistent with signaling at the cell surface rather than continued signaling following internalization. It is possible that GPR3 might not signal from intracellular sites and would need to be regulated in a manner similar to ligand-activated GPCRs in order to prevent excessive intracellular levels of cAMP. In support of this, it has been shown recently that GPR3 can interact with β-arrestin [32]. Here, we investigated the localization and possible regulation of GPR3-cAMP signaling in cultured mammalian cells. GPCRs, in general, are difficult to study because native expression levels are very low. Also, biochemical experiments with mouse oocytes are unfeasible due to the limited amount of sample that can be obtained. Therefore, we used an overexpression system in HEK293 cells, which have been commonly used to study signaling and regulation of a variety of GPCRs, to study GPR3 localization and signaling. We found that GPR3 signals at the cell surface, undergoes endocytosis, and is susceptible to desensitization by GRK2 and β-arrestin-2 overexpression. We chose to specifically study GRK2 and β-arrestin-2 because GRK2 is primarily expressed in oocytes and the oocyte only expresses β-arrestin-2 [31]. Mutations of the serine and threonine residues in the third intracellular loop markedly increased cAMP levels but these residues are not involved in desensitization by GRK2 and β-arrestin-2. Mutations of serines in the C-terminus did not affect cAMP production. Both the ST/A and S1-6A mutations decreased GPR3 surface localization, suggesting that these residues are important for receptor expression at the plasma membrane.

## Materials and Methods

### Cell Culture and Transfection

HEK293 cells were cultured in Dulbecco’s modified Eagle’s (DMEM)/F-12 medium (Invitrogen, Carlsbad, CA) supplemented with 10% fetal bovine serum (Invitrogen), 100 U/mL penicillin and 100 µg/mL streptomycin (Invitrogen) at 37°C in a humidified 5% CO_2_/95% air incubator. Cells plated in 6-well dishes were transfected with plasmid DNA using Lipofectamine 2000 (Invitrogen) following the manufacturer’s recommendations. Experiments were performed 24 hr after transfection. Bisindolylmaleimide I (Bis I) and Phorbol 12-myristate 13-acetate (PMA) were purchased from Calbiochem (La Jolla, CA) and Tocris Bioscience (Minneapolis, MN), respectively. Methyl-β-cyclodextrin was purchased from Sigma (St. Louis, MO).

### Plasmids

Mouse GPR3-RFP was provided by Y. Saeki (Ohio State University) in pHGCX Human GPR3 in pCMV6-AC-HA-His (GPR3-HA) was purchased from Origene (Rockville, MD). Dynamin 1 wildtype (WT) and K44A in pcDNA3.1 were purchased from ATCC (Manassas, VA). GRK2 was provided by R. Lefkowitz (Duke University Medical Center) in pRK5. β-arrestin-2-GFP was obtained from M. Caron (Duke University Medical Center) in pS65T and was subcloned into pSP64.5. pcDNA3.1 empty vector was purchased from Invitrogen. CFP-Epac1(δDEP-CD)-YFP was provided by K. Jalink (The Netherlands Cancer Institute).

### Transferrin Assay

Cells were washed in RPMI-1640 without bicarbonate (Invitrogen) +0.2% BSA (Sigma), pH 7.4, for 30 min at 37°C. Cells were incubated with 50 µg/mL Alexa Fluor 488-labelled transferrin (Invitrogen) for 2 min at 37°C, placed on ice for 10 min, and washed with cold PBS. Cells were fixed in 2% formaldehyde (Sigma) for 30 min at room temperature, washed twice with PBS, and transferrin internalization was examined with a Zeiss LSM 510 confocal microscope. Fluorescence was excited at 488 nm and detected at 530 nm. Images were collected using a 40X NA 1.2 water immersion objective (C-Apochromat; Carl Zeiss MicroImaging, Inc., Thornwood, NY).

### Biotinylation

Biotinylation was performed on cells in suspension according to the manufacturer’s recommendation. Cells were plated in 6-well dishes and transfected as described previously. Cells were trypsinized using 0.025% Trypsin-EDTA, resuspended in growth medium, washed three times with ice cold PBS (pH 8.0), incubated in 0.5 mg/mL sulfo-NHS-LC-Biotin (Pierce Biotechnology, Rockford, IL) for 30 min at 4°C, and washed with ice cold PBS (pH 8.0) +100 mM glycine to remove unbound biotin. For internalization assays, cell surface proteins were biotinylated with cleavable sulfo-NHS-SS-biotin (Pierce) as indicated above. Cells were then incubated in regular medium at 37°C for 30 min or 1 hr to allow internalization. Cleavage of surface biotin was performed as previously described [33]. Briefly, cells were incubated at 4°C with 3 washes of glutathione strip buffer (50 mM glutathione, 75 mM NaCl, 75 mM NaOH, 10% FBS in H_2_0) for 15 min each. Glutathione was quenched by 3 washes of iodoacetamide buffer (50 mM iodoacetamide, 1% BSA in PBS, pH 7.4) for 15 min each at 4°C.

Protein samples were prepared in RIPA buffer (Teknova, Hollister, CA) containing protease inhibitor cocktail (Pierce Biotechnology), sonicated, and rotated at 4°C for 30 min. Supernatants were collected following centrifugation at 14,000 × g for 20 min at 4°C. Protein concentrations were determined by BCA assay (Pierce Biotechnology).

### Antigen Precipitation

Cell surface biotinylated proteins were precipitated from cell lysates using streptavidin agarose resin (Pierce). Sixty µg of protein was added to 30 µl of agarose beads in a total volume of 200 µl with RIPA buffer. Samples were rotated for 2 hr at 4°C, spun at 1150×g for 5 min at 4°C, and washed four times with 500 µl RIPA buffer. After the final wash, the beads were resuspended in Laemmli sample buffer with 5% 2-Mercaptoethanol and boiled for 5 min. Five µl of the sample was analyzed by Western Blot. 0.5 µg of total lysate was used to examine total GPR3 expression and GAPDH or β-actin as a loading control. The same sample was used to examine surface and total GPR3.

### Western Blot Analysis

Western blot analysis was performed as described previously [34]. Proteins were resolved on a 4–20% Tris-HCl ready gel (Biorad, Hercules, CA), transferred onto a nitrocellulose membrane (Amersham Biosciences, Piscataway, NJ), and blocked in 5% milk for 30 min. The membrane was incubated in primary antibody overnight at room temperature, washed, incubated in horseradish peroxidase-conjugated secondary antibody for 1 hr, and washed. Primary antibodies were anti-HA high affinity (Roche Applied Science, Indianapolis, IN), GAPDH, and β-actin (Abcam, Cambridge, MA) and were diluted 1∶1000 in blocking buffer. Anti-dynamin 1/2 (Santa Cruz Biotechnology, Santa Cruz, CA) was diluted 1∶200 in blocking buffer. Secondary antibodies were obtained from Santa Cruz and diluted 1∶5000 in blocking buffer. Immunoreactive protein bands were detected using ECL Prime or ECL plus Western blotting detection system and visualized with GE Hyperfilm (Amersham Biosciences). Films were scanned and band intensities were analyzed by densitometry using Image J software (National Institutes of Health). When total GPR3 expression was examined, densitometry values were normalized to GAPDH or β-actin. To examine the amount of surface GPR3 compared to total GPR3 expression, the densitometric value for surface GPR3 was divided by the densitometric value for total GPR3 and then the ratio was normalized to GAPDH or β-actin. The samples for surface GPR3 and total GPR3 were from the same lysate.

### EIA cAMP Measurement

HEK293 cells were transfected with the indicated plasmid DNA using Lipofectamine 2000. Twenty-four hr after transfection, cells were trypsinized as described above and 400,000 cells were washed with PBS, lysed in 225 µl of 0.1 M HCl for 10 min at room temperature, sonicated, and stored at −80°C until the assay was performed. Levels of intracellular cAMP were measured using the cAMP enzyme immunoassay (EIA) kit, Direct (Sigma, or Enzo Life Sciences, Plymouth Meeting, PA), using the non-acetylation EIA procedure. Similar results were obtained using both EIA kits. Cyclic-AMP was measured from ∼170,000 cells in 100 µl of 0.1 M HCl in all experiments unless otherwise noted. Results are presented as pmol/mL of cAMP.

### Fluorescence Resonance Energy Transfer (FRET) Measurement of cAMP

To study changes in intracellular cAMP by FRET, HEK293 cells were plated on round glass cover slides and transfected 24 hr later with the CFP-Epac1(δDEP-CD)-YFP [35] sensor plasmid and, when indicated, with GPR3-RFP, Dynamin WT or K44A, GRK2 and β-arrestin-2 plasmids. 24–36 hr after transfection, cells were washed once and maintained in a physiological buffer containing 144 mM NaCl, 5.4 mM KCl, 2 mM CaCl_2_, 1 mM MgCl_2_, and 20 mM HEPES, pH 7.4 at room temperature, and placed on a Zeiss Axio Observer A1 microscope equipped with Plan-Apochromat 63×/1.4 oil immersion objective, Polychrome V light source, DV2 DualView beam splitter and CoolSNAP-HQ2 CCD-camera (Visitron Systems, Pullheim, Germany). The YFP/CFP emission ratio upon 436 nm excitation (filters YFP 535±15 nm, CFP 480±20 nm) was measured before and after saturation of the sensor with isoproterenol plus isobutylmethyl-xanthine (IBMX) or forskolin plus IBMX. Measurements were monitored online and recorded by the VisiView software (Visitron). After each measurement, emission values were corrected for bleedthrough of CFP into YFP channel and for photobleaching as described [36]. The data were analyzed with Excel and Origin 8.5 (OriginLab) packages to calculate percent change in YFP/CFP ratio. To convert FRET ratio data into absolute cAMP concentrations, the maximal response of the sensor in each cell stimulated with isoproterenol plus IBMX or with forskolin plus IBMX (ΔFRET) was measured. Next, the degree of sensor activation at basal state was calculated as (55.4 -ΔFRET)/55.4*100%, where 55.4 (±0.9, mean ± SEM) is the maximal ΔFRET measured in empty vector-transfected cells. The degree of sensor activation (y) was used to calculate cAMP concentrations (x) based on the published concentration-response-dependence measured *in vitro* [35] and the previously established protocol and equation [36], [37], [cAMP] = EC_50 *_ {(y-R_min_)/(R_max_-y)}^1/n^, where EC_50_ is the EC_50_-value of the sensor for cAMP determined in vitro [35]and is equal to 13.21 µM; n is the Hill coefficient (n = 1) from the in vitro concentrations-response dependency [35]; R_min_ = 0, and R_max = _100. The resulting equation [cAMP] = 13.21*y/(100-y) which describes the published curve, was used to calculate cAMP concentrations. Values are presented as mean ± SEM.

### Mutagenesis

GPR3-HA mutants, in which serine and threonine residues in the third intracellular loop and C-terminus were mutated to alanine, were generated using the QuikChange II site-directed mutagenesis Kit (Agilent, Cedar Creek, TX) according to the manufacturer’s protocol. Amino acids at 237 and 242 in the third intracellular loop (S,T) and amino acids at 316, 317, 318, 324, 326, 328 in the C-terminus (1–6) were mutated to alanine. HPLC purified primers for mutagenesis were purchased from Sigma (Table 1) and mutations were made in the order the primers are presented. Three mutants were generated: GPR3-HA ST/A, GPR3-HA S1-6A, GPR3-HA ST/A+S1-6A. A GRK2-K220R catalytically inactive mutant where the lysine at position 220 was mutated to arginine was made using the QuikChange II site-directed mutagenesis kit and the primers: 5′CAAGATGTACGCCA-TGAGGTGTCTGGACAAGAAGC 3′ and 5′ GCTTCTTGTCCAGACACCTCATGGC-GTACATCTTG 3′.

**Table 1:** Primer sets used for GPR3-HA mutagenesis.

GPR3-HA S1-3A	5′-GGGCTGTCTGCTGCTGCTGATCCTCTTCCAAGATCCCCTTCC-3′ 5′-GGAAGGGGATCTTGGAAGAGGATCAGCAGCAGCAGACAGCCC-3′
GPR3-HA S6A	5′-GATCCCGCTCCCCCAGTGATGTCACGCGTT-3′ 5′-AACGCGTGACATCACTGGGGGAGCGGGATC-3′
GPR3-HA S4,5A	5′-GATCCCCTTCCGAGCCCGCGCCCCCGCTGATG-3′ 5′-CATCAGCGGGGGCGCGGGCTCGGAAGGGGATC-3′
GPR3-HA T-A	5′-CACTATGTGGCCGCACGCAAGGGCATT-3′ 5′-AATGCCCTTGCGTGCGGCCACATAGTG-3′
GPR3-HA S-A	5′-CCTGCTGCCTGCCGCCCACTATGTG-3′ 5′-CACATAGTGGGCGGCAGGCAGCAGG-3′

### Statistical Analysis

Data are representative of at least three independent experiments, unless otherwise specified. Values were analyzed by One-way ANOVA, Repeated Measures ANOVA, or Student’s *t* test as described in each figure legend. Statistical analysis was performed using Graph Pad Prism software and significance was assessed at *P*<0.05.

## Results and Discussion

### Inhibition of Endocytosis in HEK293 Cells by Overexpressing a Dominant Negative form of Dynamin (Dyn K44A) Increases Cell Surface Localization of GPR3

**Figure 1:**
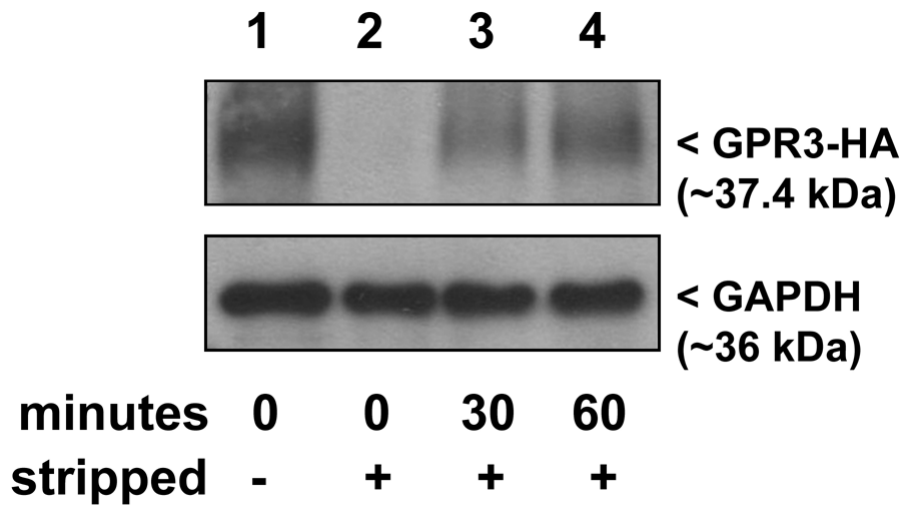
GPR3 is present at the cell surface and undergoes internalization. HEK293 cells were transfected with GPR3-HA. Twenty-four hr later, surface proteins were biotinylated at 4°C (lane 1), allowed to internalize for 30 min (lane 3) or 60 min (lane 4) at 37°C, and biotinylated proteins remaining at the cell surface were removed using a glutathione strip buffer. Biotinylated proteins were precipitated from the total lysate and 5 µl of the sample was analyzed by Western blotting. Lane 2 shows efficient stripping of biotin from the cell surface GPR3. Detection of GAPDH in 0.5 µg of total lysate was run on the same gel and served as a loading control to confirm that an equivalent amount of protein was used for precipitations. Representative blot of 2 separate experiments.

To confirm that GPR3 is endocytosed in HEK293 cells, cell surface proteins were biotinylated with cleavable sulfo-NHS-SS-biotin and incubated at 37°C to allow for internalization. Following internalization, biotinylated proteins remaining at the cell surface were stripped and internalized biotinylated proteins were detected by streptavidin precipitation and Western blot. Biotinylated GPR3 was detected at the cell surface (Lane 1 of Fig. 1) and in internalized samples at 30 min and 60 min (Lane 3 and 4 of Fig. 1). The internalized sample did not contain cell surface GPR3 since efficient stripping of biotinylated cell surface proteins was observed (Lane 2 of Fig. 1).

**Figure 2:**
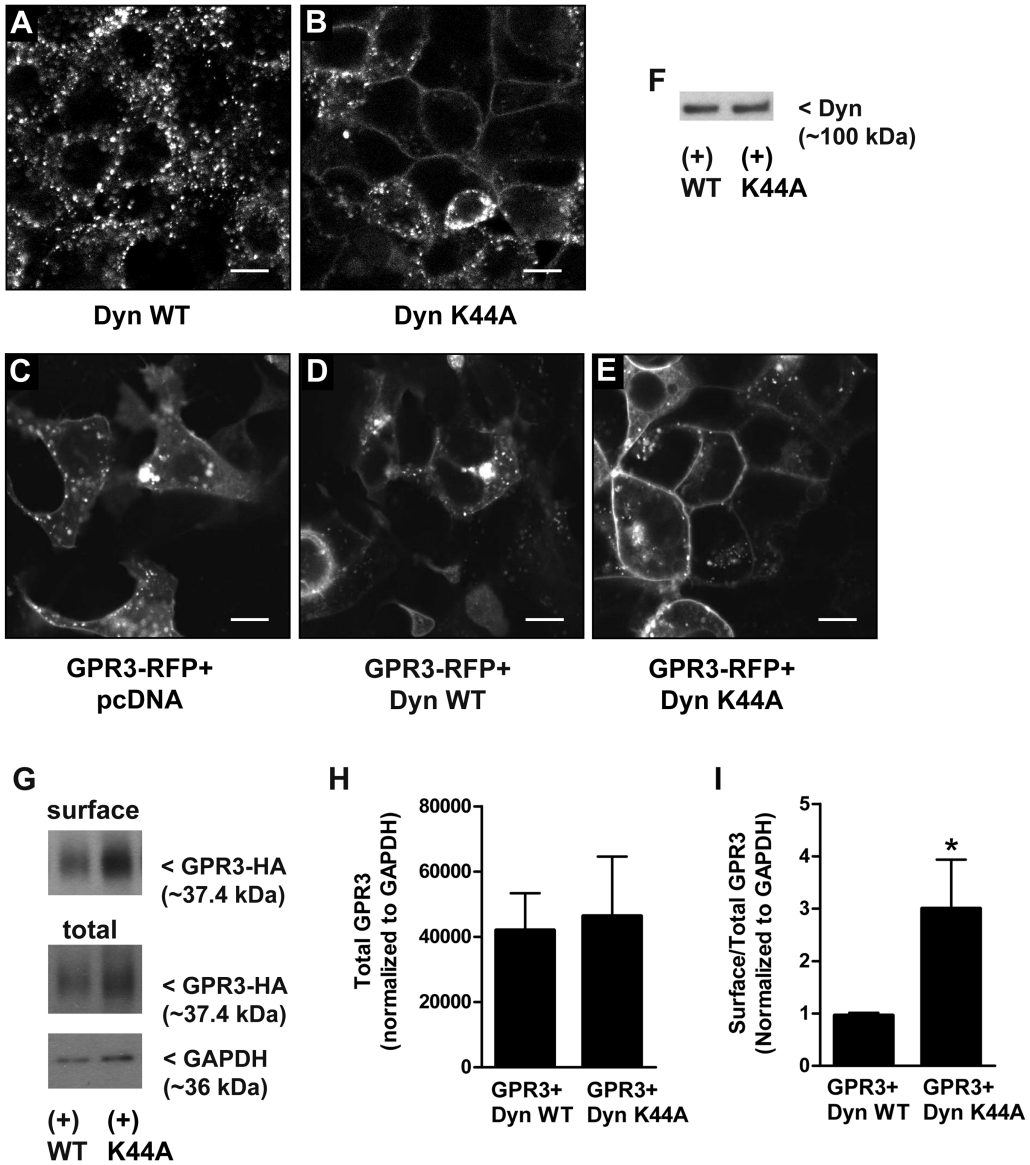
Inhibition of endocytosis increases cell surface GPR3. (**A and B**) HEK293 cells were transfected with Dyn WT (**A**) or Dyn K44A (**B**) and 24 hr later, the cells were incubated with Alexa Fluor 488-labelled transferrin and imaged with a confocal microscope. Images are representative of 3 separate experiments. (**C–E**) Cells were co-transfected with GPR3-RFP and pcDNA (**C**), or Dyn WT (**D**) or Dyn K44A (**E**) and imaged using a confocal microscope 24 hr later. Bar = 10 µm. (**F**) One µg of HEK293 lysate was used to detect Dyn WT and Dyn K44A overexpression by Western blot. (**G**) HEK293 cells were co-transfected with GPR3-HA and Dyn WT or Dyn K44A and 24 hr after transfection cell surface proteins were biotinylated, precipitated, and surface expression of GPR3-HA was detected by Western blot analysis. 0.5 µg of total lysate was used to detect total GPR3 and GAPDH expression. Blot is representative of 3 separate experiments. (**H–I**) Bands corresponding to surface GPR3 and total GPR3 expression were analyzed using densitometry. **H**) Densitometric values for total GPR3 expression in cell lysates were normalized to GAPDH. **I**) The densitometric value for surface GPR3 was divided by the densitometric value for total GPR3 expression and normalized to GAPDH to compare the amount of GPR3 at the surface vs. total GPR3 expression. (*) indicates a significant increase in cell surface GPR3 compared to “GPR3+Dyn WT”. Significance was determined by Student’s *t* test (*p*<0.05). Results are presented as mean ± S.E.M from 3 separate experiments.

In order to examine whether GPR3 signals from the cell membrane and/or endosomes, we used Dyn K44A to inhibit endocytosis [38]. Dyn K44A is mutated in the GTP binding domain which blocks early events of endocytosis. To confirm that Dyn K44A effectively inhibits endocytosis in HEK293 cells, Dyn WT and K44A were expressed by transient transfection and 24 hr later, the cells were incubated with Alexa Fluor 488-labeled transferrin to evaluate receptor-mediated internalization. Confocal images demonstrate that cells transfected with Dyn K44A have lower levels of transferrin internalization with more accumulation at the cell surface compared to cells transfected with dynamin WT (Dyn WT) (Fig. 2A and B). These results are consistent with previous findings that Dyn K44A inhibits receptor-mediated endocytosis [38].

Localization of GPR3 in response to endocytic inhibition was evaluated using two methods. First, HEK293 cells were co-transfected with GPR3-RFP and pcDNA, or Dyn WT or Dyn K44A, and imaged by confocal microscopy. In cells co-transfected with pcDNA or Dyn WT, GPR3-RFP is localized in the plasma membrane and also within intracellular clusters (Fig. 2C and 2D), similar to its localization in mouse oocytes [31] and transfected Neuro2a cells [15]. Co-transfection of GPR3-RFP with Dyn K44A increased GPR3 fluorescence at the plasma membrane (Fig. 2E). Western blot analysis confirmed that Dyn WT and Dyn K44A were expressed at equivalent levels (Fig. 2F). Second, GPR3-HA localization at the plasma membrane was evaluated by biotinylation of cell surface proteins, precipitation from cell lysates, and Western blot analysis (Fig. 2G). Bands observed for surface GPR3 and total GPR3 were analyzed by densitometry. We found that total GPR3 expression was similar in cells co-transfected with GPR3-HA and Dyn WT or Dyn K44A (Fig. 2H). Next, we compared the ratio of surface GPR3 vs. total GPR3 expression. Consistent with the results from confocal microscopy, there was a ∼3 fold increase in surface GPR3 precipitated from biotinylated cell lysates co-transfected with Dyn K44A (∼3.0±0.93, n = 4) compared to Dyn WT (∼0.96±0.05, n = 3) (Fig. 2 I). Cell surface GPR3 and total GPR3 expression was similar in cells co-transfected with pcDNA or Dyn WT (data not shown); therefore, only densitometry data for Dyn WT is shown. An increase in cell surface GPR3-HA was also observed when transfected cells were treated with methyl-β-cyclodextrin (MβCD), a drug that selectively extracts cholesterol from the plasma membrane and inhibits receptor-mediated endocytosis by preventing invagination of caveolae and clathrin-coated pits [39] (data not shown). These results demonstrate that GPR3 is localized at the cell surface and is internalized in HEK293 cells.

### Inhibition of Endocytosis in Cells Transfected with GPR3-HA Increases Intracellular cAMP Levels

**Figure 3:**
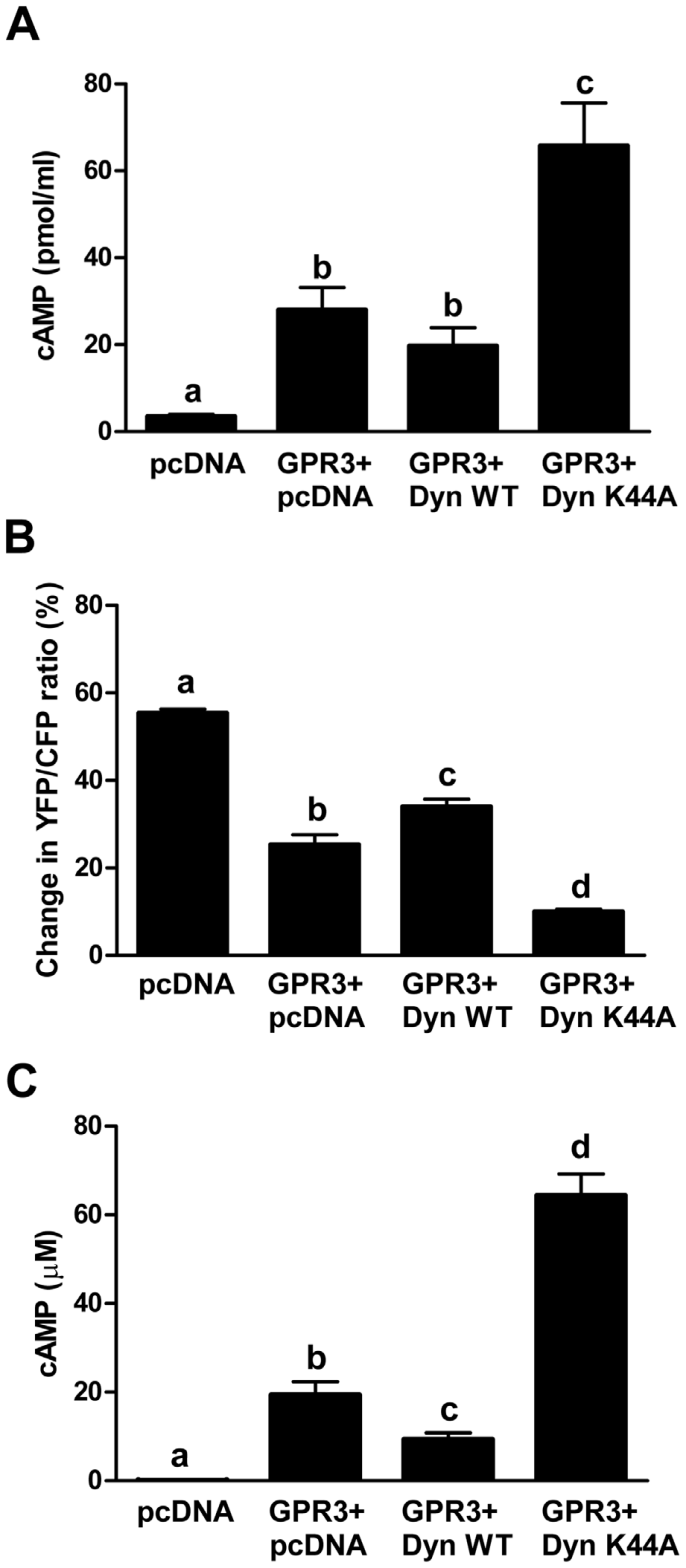
Inhibition of endocytosis increases intracellular cAMP. **A)** HEK293 cells were co-transfected with GPR3-HA and pcDNA or with Dyn WT or Dyn K44A. Twenty-four hr after transfection, cells were harvested for cAMP EIA. Bars with different letters are significantly different. Significance was determined by One-way ANOVA followed by Newman-Keuls multiple comparison test (*p*<0.01).Results are presented as mean ± S.E.M from 4 separate experiments. **B–C**) HEK293 cells were co-transfected with GPR3-RFP and CFP-Epac1(δDEP-CD)-YFP and pcDNA, or Dyn WT or Dyn K44A. The YFP/CFP ratios were measured before and after isoproterenol and IBMX treatment. **B)** Percent change in the YFP/CFP ratio. **C**) FRET measurements were converted to cAMP levels (µM) as described in the experimental protocol. Bars with different letters are significantly different. Significance was determined by One-way ANOVA followed by Newman-Keuls Multiple Comparison Test (*p*<0.01). Results were obtained from 15 different GPR3-RFP expressing cells per group.

GPCR internalization usually leads to signal termination. However, recent studies have shown that several receptors including TSH and PTH receptors continue to signal following internalization [24]–[26]. In these previous studies, cAMP levels returned toward basal, unstimulated levels following ligand treatment and endocytic inhibition. Because constitutively active GPCRs signal in the absence of an agonist, it is possible that placement at the plasma membrane is not required for G protein activation and that they may be able to signal internally. To determine if GPR3 signals following endocytosis, we measured cAMP using an EIA assay in cells co-transfected with GPR3-HA and pcDNA, or Dyn WT or K44A. Transient transfection of HEK293 cells with GPR3-HA and pcDNA caused cAMP levels to increase significantly compared to pcDNA empty vector-transfected cells, confirming the constitutive activity of GPR3. When co-transfected with Dyn K44A, cAMP levels significantly increased compared to cells co-transfected with pcDNA or Dyn WT (Fig. 3A). Thus, the increase in GPR3 at the cell surface corresponds to an increase in cAMP levels and demonstrates that GPR3 signals at the cell surface and not from endosomes. Transfection of Dyn WT or K44A in the absence of GPR3-HA did not alter cAMP levels (data not shown). Additionally, treatment of GPR3-HA transfected cells with MβCD, which prevents endocytosis and increases surface GPR3, also significantly increased cAMP levels (data not shown).

In order to corroborate the cAMP data obtained by EIA assay, we performed a FRET-based assay using a low affinity (K_d_ = ∼14 µM) Epac-based cAMP sensor (CFP-Epac1(δDEP-CD)-YFP [35]), which is not saturated in cells transfected with GPR3-RFP. This method is advantageous over standard EIA methods since only transfected cells are used for cAMP measurements. Cells were co-transfected with GPR3-RFP, Epac sensor, and pcDNA or Dyn WT or Dyn K44A. GPR3-positive red cells of similar intensity were selected for all recordings. The YFP/CFP emissions were recorded before and after isoproterenol/IBMX treatment to calculate the percent change in FRET (Fig. 3B) and FRET ratios were converted into absolute cAMP concentrations (Fig. 3C). As expected, cells transfected with GPR3-RFP have a lower percent change in FRET and higher levels of cAMP (∼19 µM) compared to cells transfected with pcDNA (∼0.1 µM). Cells co-transfected with GPR3-RFP and Dyn K44A have a lower percent change in FRET and higher cAMP levels (∼64 µM) compared to cells co-transfected with GPR3-RFP with Dyn WT (∼9 µM) or pcDNA(Fig. 3B). This result confirms that cAMP levels significantly increase when endocytosis is inhibited and cell surface GPR3 expression is increased. Using this method, we also detected a significant decrease in cAMP in cells co-transfected with GPR3 and Dyn WT compared to GPR3 and pcDNA. It is possible that overexpression of Dyn WT increases GPR3 endocytosis and therefore decreases cAMP production, although we did not detect a difference in GPR3-HA membrane localization when cells were co-transfected with pcDNA or Dyn WT (data not shown). Together, these results demonstrate that GPR3 signals at the cell surface and does not signal from endosomal compartments. This finding is in contrast to GPR6, a closely related receptor to GPR3, which is thought to signal internally because it is primarily localized in intracellular compartments [40]. GPR3 seems to be largely localized on the cell surface in oocytes [31], Neuro2a cells [15], and HEK293 cells [41] and this difference in localization between GPR3 and GPR6 may explain why they may signal from different membrane compartments.

### Overexpression of GRK2 and β-arrestin-2 Decreases Surface Localization of GPR3 and cAMP Production

**Figure 4:**
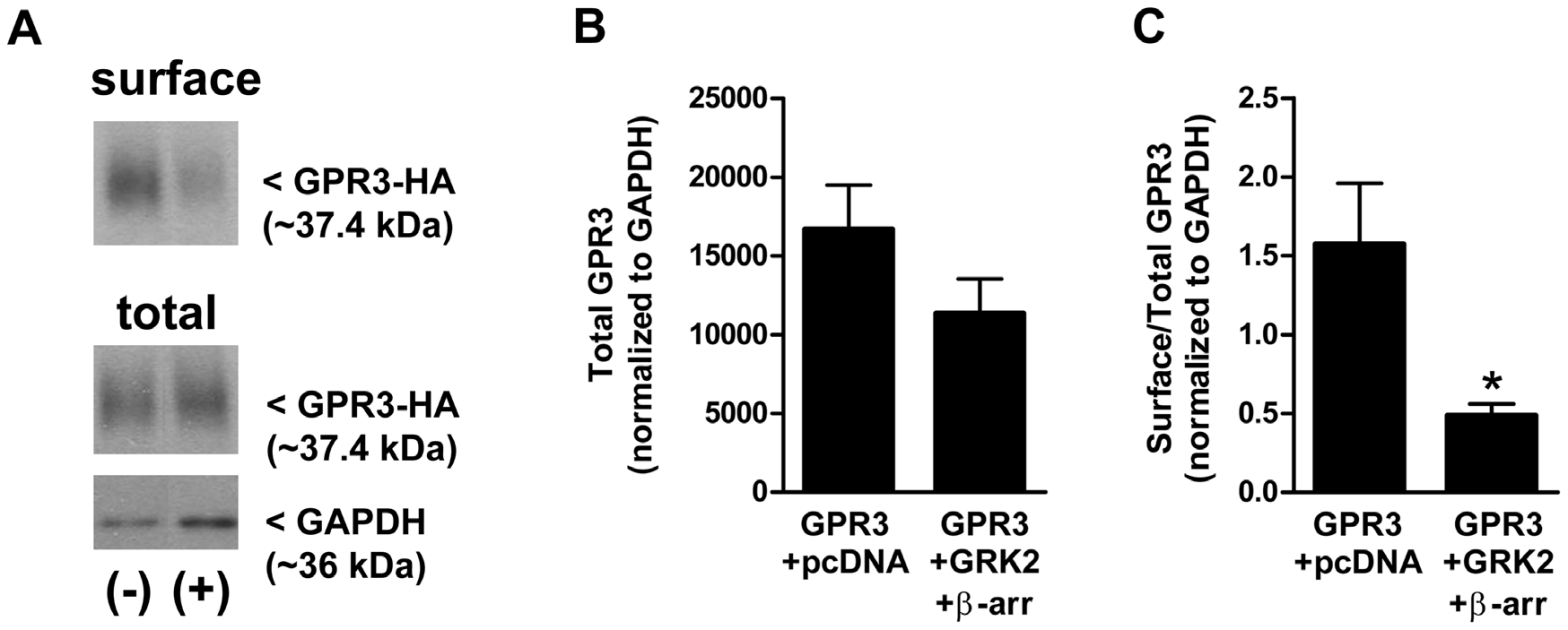
Overexpression of GRK2 and β-arrestin-2 decreases cell surface GPR3 expression. **A**) HEK293 cells were co-transfected with GPR3-HA and pcDNA (**−**) or GPR3-HA and GRK2 and β-arrestin-2 (**+**). Twenty-four hr after transfection, cell surface proteins were biotinylated, precipitated, and cell surface expression of GPR3-HA was detected by Western blotting. 0.5 µg of total lysate was used to detect total GPR3 and GAPDH expression. Blot is representative of 3 separate experiments. **B–C**) Bands corresponding to surface GPR3 and total GPR3 expression were analyzed using densitometry. Densitometric values for total GPR3 expression were normalized to GAPDH. Densitometric values for surface GPR3 was divided by densitometric values for total GPR3 and normalized to GAPDH to compare the amount of GPR3 at the surface vs. total GPR3 expression. (*) indicates a significant decrease in surface/total GPR3 expression as a result of GRK2 and β-arrestin-2 overexpression. Significance was determined by Student’s *t* test (*p*<0.05). Results are presented as mean ± S.E.M. from 3 separate experiments.

**Figure 5:**
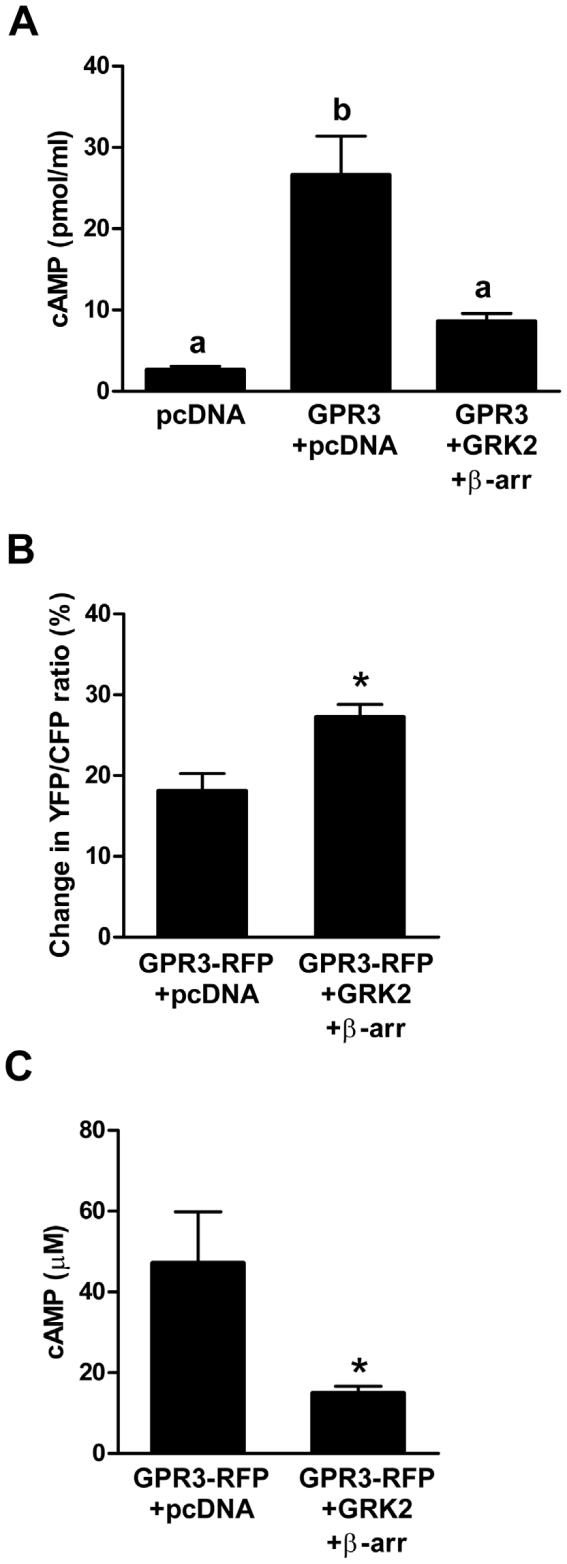
Overexpression of GRK2 and β-arrestin-2 decreases intracellular cAMP levels. **A**) HEK293 cells were co-transfected with GPR3-HA and pcDNA or GPR3-HA and GRK2 and β-arrestin-2. Twenty-four hr after transfection, cells were harvested for cAMP EIA. Bars with different letters are significantly different. Significance was measured by One-way ANOVA followed by Newman-Keuls Multiple Comparison Test (*p*<0.05). Results are presented as mean ± S.E.M from 3 separate experiments. **B–C**) HEK293 cells were co-transfected with GPR3-RFP, CFP-Epac1(δDEP-CD)-YFP, and pcDNA or GPR3-RFP, CFP-Epac1(δDEP-CD)-YFP, GRK2, and β-arrestin-2. The YFP/CFP ratios were measured before and after forskolin and IBMX treatment. **B)** Percent change in the YFP/CFP ratio. **C**) FRET measurements were converted to cAMP levels (µM) as described in the experimental protocol. (*) indicates a significant difference in the % change in YFP/CFP ratio or cAMP levels compared to “GPR3-RFP+pcDNA”. Significance was determined by Student’s *t* test (*p*<0.05). Results were obtained from 13–18 different GPR3-RFP expressing cells per group.

It is unknown whether GPR3 is regulated by similar mechanisms as ligand-activated GPCRs. It is thought that constitutively active GPCRs are in the appropriate conformation to be continuously phosphorylated by GRKs and signaling is transiently silenced [42]. To determine if GPR3 can be regulated by GRKs or β-arrestins, these proteins were co-expressed with GPR3 and localization was evaluated by the same methods described above. Overexpression of GRK2 and β-arrestin-2 significantly decreased GPR3-HA cell surface localization as assessed by biotinylation, precipitation, and Western blot analysis (Fig. 4A). Total GPR3 expression was similar in both groups (Fig. 4B). However, surface GPR3 expression compared to total GPR3 expression was significantly lower in cells co-transfected with GRK2 and β-arrestin-2 (Fig. 4C), indicating that consistent with their action on other GPCRs, these proteins induce GPR3 internalization. Measurements of cAMP using EIA and FRET showed that cAMP levels decreased by more than 50% as a result of GPR3-HA co-expression with GRK2 and β-arrestin-2 (Fig. 5 A, B and C).

These results demonstrate that GPR3 is susceptible to desensitization by a GRK2- and β-arrestin-2-dependent mechanism. There is an example of at least one constitutively active GPCR, the human cytomegalovirus (HCMV)-encoded receptor US28, which signals at the cell surface and is constitutively phosphorylated by GRKs. Following phosphorylation, US28 is rapidly internalized through a clathrin-mediated mechanism, which may not be dependent on β-arrestins [43]–[45], and internalization attenuates US28-induced G_q_-mediated phospholipase signaling [44]–[47]. Deletion or mutation of the C-terminus of US28 so that it can no longer be phosphorylated results in an increase in phospholipase signaling and an increase in surface expression [44], [45], [48]. It has been speculated that this behavior allows HCMV to evade the immune system and contributes to viral infection. Whether all constitutively active GPCRs are regulated in a similar manner or whether this is unique to US28 is not understood. GPR3 seems to be similar to US28 in that internalization induced by GRK2 and β-arrestin overexpression attenuates cAMP signaling. Whether GPR3 is regulated by this pathway under physiological conditions remains to be shown.

### The Serine and Threonine Residues in the Third Intracellular Loop and C-terminus Regulate GPR3 Activity and Surface Localization

**Figure 6:**
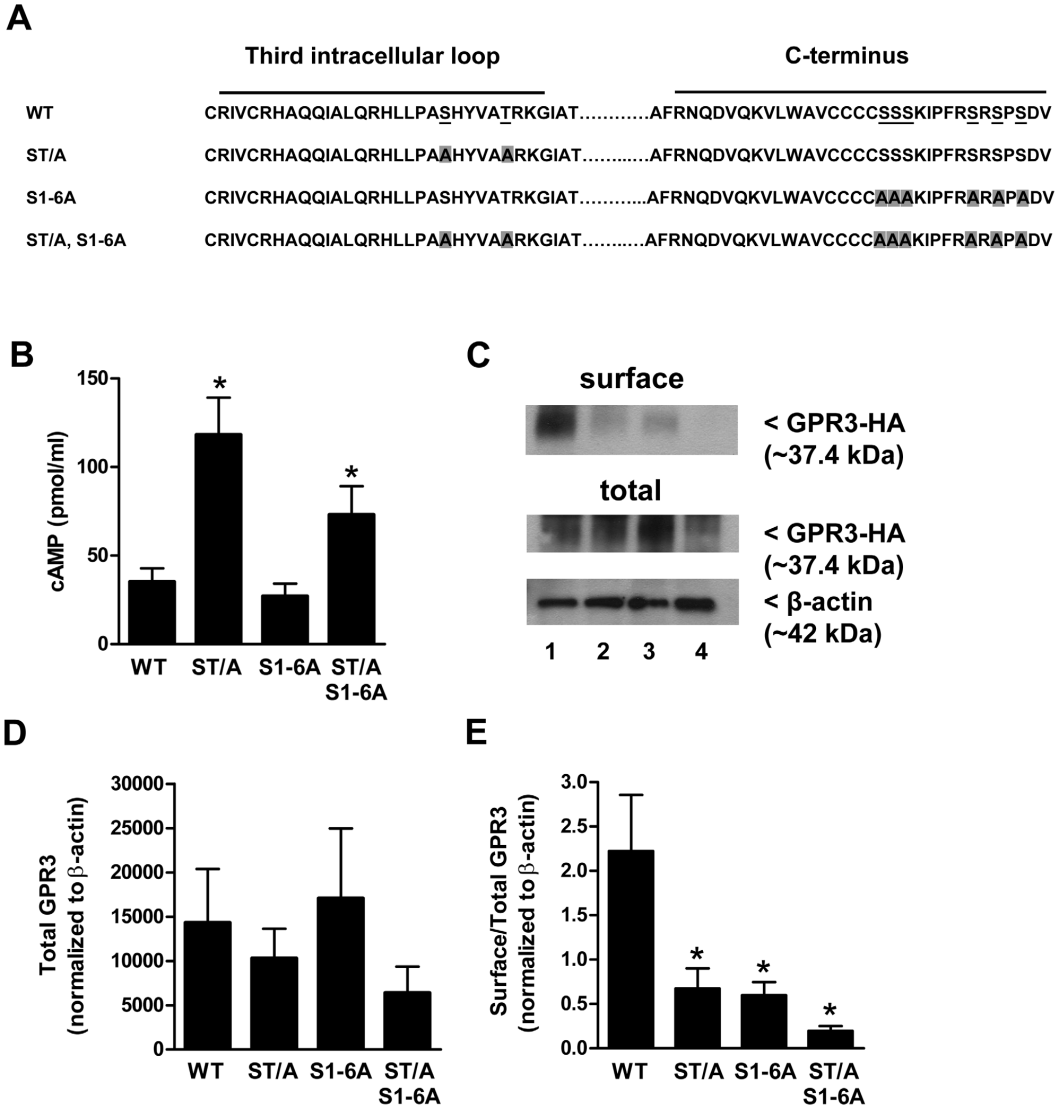
Mutation of S and T residues in the third intracellular loop increases intracellular cAMP. **A**) Schematic identifying potential serine and threonine residues in the third intracellular loop and C-terminus that could be targeted for regulation by phosphorylation. These residues were mutated to alanine to create 3 mutants: ST/A, S1-6A, and ST/A+S1-6A. **B**) GPR3-HA WT and mutants were transfected into HEK293 cells and harvested 24 hr later for cAMP EIA. (*) indicates a significant increase in cAMP level compared to “WT”. Significance was determined by Repeated Measures ANOVA followed by Dunnett’s Multiple Comparison Test (*p*<0.01). Results are presented as mean ± S.E.M. from 6 separate experiments. **C)** 24 hr after transfection, cell surface proteins were biotinylated, precipitated, and surface expression of GPR3-HA was detected by Western blotting. 0.5 µg of total lysate was used to detect total GPR3 and β-actin expression. Blot is representative of 3 separate experiments: WT, lane 1; ST/A, lane 2; S1-6A, lane 3; ST/A+S1-6A, lane 4. **D–E**) Bands corresponding to surface GPR3 and total GPR3 expression were analyzed using densitometry. Densitometric values for total expression of GPR3 WT and mutants were normalized to β-actin (**D**). The densitometric value for surface GPR3 was divided by the densitometric value for total GPR3 expression and normalized to β-actin to compare the amount of GPR3 at the surface vs. total GPR3 (**E**). (*) indicates a significant decrease in surface expression of GPR3 mutants compared to “WT”. Significance was determined by One-way ANOVA followed by Newman-Keuls Multiple Comparison Test (*p*<0.05). Results are presented as mean ± S.E.M from 3 separate experiments.

GPR3 contains two potential sites for phosphorylation by protein kinase C (PKC) as well as several serine residues that could be targets for GRKs. To determine if GPR3 activity is regulated by any of these sites, we mutated all six serines in the C-terminus and the serine and threonine residues in the third intracellular loop to alanine (Fig. 6A). If GPR3 is desensitized due to the phosphorylation of these residues, then it is expected that mutations of these sites would prevent desensitization and increase cAMP levels. We found that mutation of the six serines in the C-terminus (S1-6A) produced cAMP levels comparable to GPR3 WT. In contrast, mutation of the serine and threonine residues in the third intracellular loop (ST/A) produced significantly higher cAMP levels (Fig. 6B), suggesting that these residues are important for phosphorylation and desensitization of GPR3. The total expression of the GPR3 WT and mutants varied but were not significantly different (Fig. 6D). Therefore, it is unlikely that differences in cAMP production are due to differences in receptor expression. It is expected that if the ST/A mutant is unable to be phosphorylated and resistant to desensitization, internalization would be impaired, and surface localization would increase. However, using cell surface biotinylation, precipitation, and Western blot analysis (Fig. 6 C-E) we found that surface localization of the ST/A, S1-6A, and ST/A+S1-6A mutants was significantly lower compared to GPR3 WT (Fig. 6 E). These findings are in contrast to a recent study that reported similar cellular distribution of GPR3 WT and a C-terminal serine mutant in CHO-K1 cells [32].

Together, these results demonstrate that the ST/A and S1-6A residues have different roles for cAMP signaling but both are important for surface expression of GPR3. It is not understood why the mutants exhibit lower surface expression. It is possible that the mutated receptors do not make it to the membrane as efficiently as the WT. Because decreased cell surface expression of the mutants does not prevent the receptor from producing cAMP, it suggests that either surface localization is not a requirement for cAMP production or that there is enough of the mutated receptor at the plasma membrane to produce cAMP at comparable levels to GPR3 WT. Although our previous findings support the hypothesis GPR3 does not signal following internalization, it is conceivable that some GPR3 may be able to signal internally prior to plasma membrane insertion. There is evidence that GPCRs preassemble with signaling components before reaching the cell surface. For example, the β_2_-adrenergic receptor (β_2_AR) forms a complex with G_s_ and adenylate cyclase soon after leaving the ER in transit to the Golgi [49], [50]. However, β_2_AR does not activate G_s_ at this compartment because it does not interact with its ligand until it reaches the cell surface. Since GPR3 is thought to signal in the absence of a ligand, it is possible that it could signal internally prior to membrane insertion; however, this has not been directly examined. It has been shown that inhibition of exocytosis in *Xenopus* oocytes results in premature meiotic resumption [51], supporting the idea that GPR3 is actively trafficked to the cell surface but does not signal during exocytosis. In HEK293 cells, GPR3 may be able to signal internally from other membranes but further studies are needed in order to understand how these residues regulate surface localization of GPR3 and what membranes GPR3 can signal from.

### The Serine and/or Threonine in the Third Intracellular Loop are not Targeted by GRK2 and β-arrestin-2 to Regulate cAMP Production

**Figure 7:**
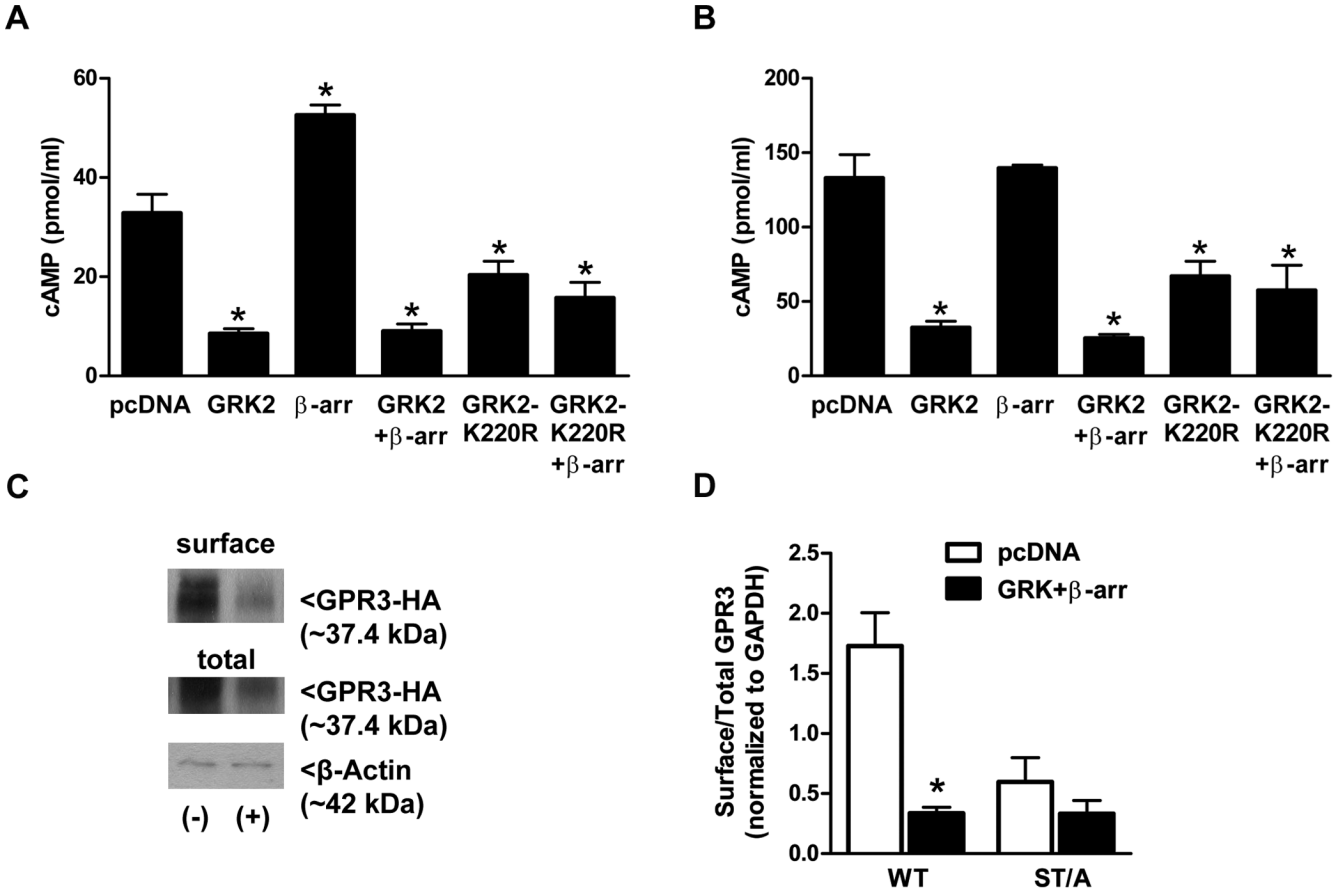
The effects of GRK2, GRK2-K220R and β-arrestin 2 on GPR3 WT and ST/A activity and surface localization. GPR3-HA WT (**A**) and ST/A (**B**) were co-transfected with pcDNA, or GRK2, or GRK2 and β-arrestin-2, or GRK2-K220R, or GRK2-K220R and β-arr-2. Twenty-four hr after transfection, cells were harvested for cAMP EIA. (*) indicates a significant difference in cAMP compared to “pcDNA”. Significance was determined by One-way ANOVA followed by Newman-Keuls Comparison Test (*p*<0.05). Results are presented as mean ± S.E.M from 3–5 separate experiments. (**C**) HEK293 cells were co-transfected with GPR3ST/A and pcDNA (−) or GRK2 and β-arrestin-2 (+). Twenty-four hr after transfection, cell surface proteins were biotinylated, precipitated from cell lysates, and analyzed by Western blotting. 0.5 µg of total lysate was used to detect total GPR3 and β-actin expression. Blot is representative of 3 separate experiments. **D**) Densitometric values for surface GPR3 was divided by densitometric value for total GPR3 and normalized to β-actin to compare the amount of GPR3 at the surface compared vs. total GPR3 expression. (*) indicates a significant decrease in surface/total GPR3 as a result of GRK2 and β-arrestin-2 expression. Significance was determined by Two-way ANOVA followed by Bonferroni Multiple Comparison Test (*p*>0.05). Results are presented as mean ± S.E.M from 3 separate experiments.

One possibility that may explain why the ST/A mutant is hyperactive is that it is resistant to desensitization. To determine if the serine and threonine residues in the third intracellular loop are required for silencing of GPR3 signaling by GRK2 and β-arrestin-2, cAMP levels were measured in cells co-transfected with the GPR3 ST/A mutant and GRK2 and β-arrestin-2. We found that cAMP levels produced by the GPR3 ST/A decreased in response to GRK2 and β-arrestin-2 overexpression. cAMP levels decreased by ∼76% and ∼73% for GPR3 WT and ST/A, respectively (Fig. 7A and B). Contrary to our expectations, these data demonstrate that mutation of potential phosphorylation sites in the third intracellular loop did not result in loss of regulation by GRK2 and β-arrestin-2 and these residues are not involved in desensitization by this pathway. We also found that surface expression of GPR3 ST/A did not significantly decrease in response to GRK2 and β-arrestin-2 overexpression (Fig. 7C and D). Perhaps there is not enough GPR3 ST/A at the cell surface to detect a more obvious change in surface localization as a result of GRK2 and β-arrestin-2 overexpression. It is also possible that the ST/A mutant is not internalized by GRK2 and β-arrestin. Further studies are required to determine the role of the third intracellular loop in the regulation of GPR3 activity. In addition to GRK phosphorylation, the third intracellular loop can regulate GPCR signaling by influencing G protein activation and binding of GPCR-interacting proteins such as 14-3-3, spinophilin, RGS2, and arrestin [52]–[56]. The third intracellular loop is also thought to be involved in stabilizing the neuropeptide Y1 receptor in the inactive state and confers structural properties for regulating receptor activation [57].

Because the ST/A mutant produced higher levels of cAMP but did not have increased cell surface expression, it raises the question of whether desensitization and internalization of GPR3 are distinct processes. To address this, we examined the individual effects of GRK2 and β-arrestin on cAMP production. We found that overexpression of GRK2 by itself is sufficient to significantly decrease cAMP production by GPR3 WT and GPR3 ST/A to the same extent as GRK2 with β-arrestin-2 (Fig. 7A and B). However, overexpression of GRK2 or β-arrestin alone did not reduce surface localization (data not shown). A significant decrease in the surface localization of GPR3 WT is only detected when both GRK2 and β-arrestin-2 are transfected together. Therefore, GPR3 activity can be silenced by GRK2 alone whereas the addition of β-arrestin is required for GPR3 internalization.

Surprisingly, overexpression of β-arrestin alone significantly increased cAMP levels for GPR3 WT (Fig. 7A) but had no effect on the ST/A mutant (Fig. 7B). This effect of β-arrestin is unlikely to be non-specific since transfection of β-arrestin without GPR3 does not increase cAMP production in HEK293 cells (data not shown). It is also not associated with increased GPR3 at the cell surface, because surface expression of GPR3-HA in cells transfected with GPR3-HA and β-arrestin was not different from cells transfected with GPR3-HA alone (data not shown). There is a recent report of β-arrestin overexpression increasing cAMP production by the PTH receptor. In response to PTH stimulation, the PTH receptor is internalized where it forms a stable complex with arrestin and βγ. This association increases the rate of G_S_ activation and the steady-state levels of activated G_S_, thereby leading to prolonged generation of cAMP from endosomal compartments [58]. GPR3 does not appear to signal from endosomes, but perhaps β-arrestin forms a stable complex with GPR3 at the cell surface and increases G_s_ activation and cAMP production. In the presence of GRK2, either this association may not occur, or the complex is internalized and desensitized. Interestingly, we did not detect an increase in cAMP when β-arrestin was overexpressed with the ST/A mutant, suggesting that an interaction between GPR3 and β-arrestin is required for cAMP production. Future studies are required to understand the role of β-arrestin in GPR3 signaling and trafficking and the importance of phosphorylation for these processes.

The GPR3 ST/A mutant is susceptible to desensitization by GRK2 and β-arrestin overexpression; therefore, it is possible that other sites within GPR3 are phosphorylated by GRK or that GRK mediates desensitization independently of phosphorylation. To test this, we constructed a catalytically inactive GRK2 mutant (GRK2-K220R), in which the lysine at residue 220 was mutated to arginine. This mutation in the ATP binding domain has been shown previously to inhibit the kinase activity of GRK2 [44], [46]. HEK293 cells were co-transfected with GPR3 WT or ST/A and GRK2-K220R and β-arrestin-2-GFP. GRK2-K220R significantly decreased cAMP for GPR3 WT and ST/A, although the decrease was not to the same extent as GRK2 WT (Fig. 7 A and B). We also examined the effect of GRK2-K220R on GPR3 localization using biotinylation. We found that overexpression of GRK-K220R by itself or with β-arrestin-2 did not change the surface localization of GPR3 WT (data not shown). This supports the idea that kinase activity of GRK2 is needed to induce GPR3 internalization, but not to diminish cAMP levels. The ability of GRK2 to desensitize GPCR activity independently of its kinase activity has been reported for other GPCRs including the metabotropic glutamate receptor, parathyroid hormone receptor, and type 1A angiotensin II [59]–[61]. The amino terminus of GRK2 contains a regulator of G-protein signaling (RGS) homology (RH) domain which can regulate G protein activation by binding to and sequestering G proteins in the cytoplasm to prevent further interactions with GPCRs (reviewed in [62]). Indeed, expression of the amino-terminal domain of GRK, which contains the RH domain, is sufficient to diminish cAMP activity without phosphorylation of several GPCRs [52], [63], [64]. Based on our studies, it is unclear whether GPR3 phosphorylation by GRKs is important for GPR3 signaling, since both wild-type and kinase-inactive GRK2 inhibited cAMP production. Although the GRK2-K220R mutant significantly decreased cAMP levels, it was not to the same extent as GRK2 WT, suggesting that phosphorylation may also be important. Future studies are needed to determine if GPR3 is phosphorylated, if this phosphorylation is diminished with the ST/A mutant, and if the RH domain of GRK2 can reduce GPR3 activity. It is also possible that other GRKs can regulate GPR3 activity in HEK293 cells.

### PKC Inhibition Increases GPR3-cAMP Signaling and PKC Activation Decreases GPR3-cAMP Signaling

**Figure 8:**
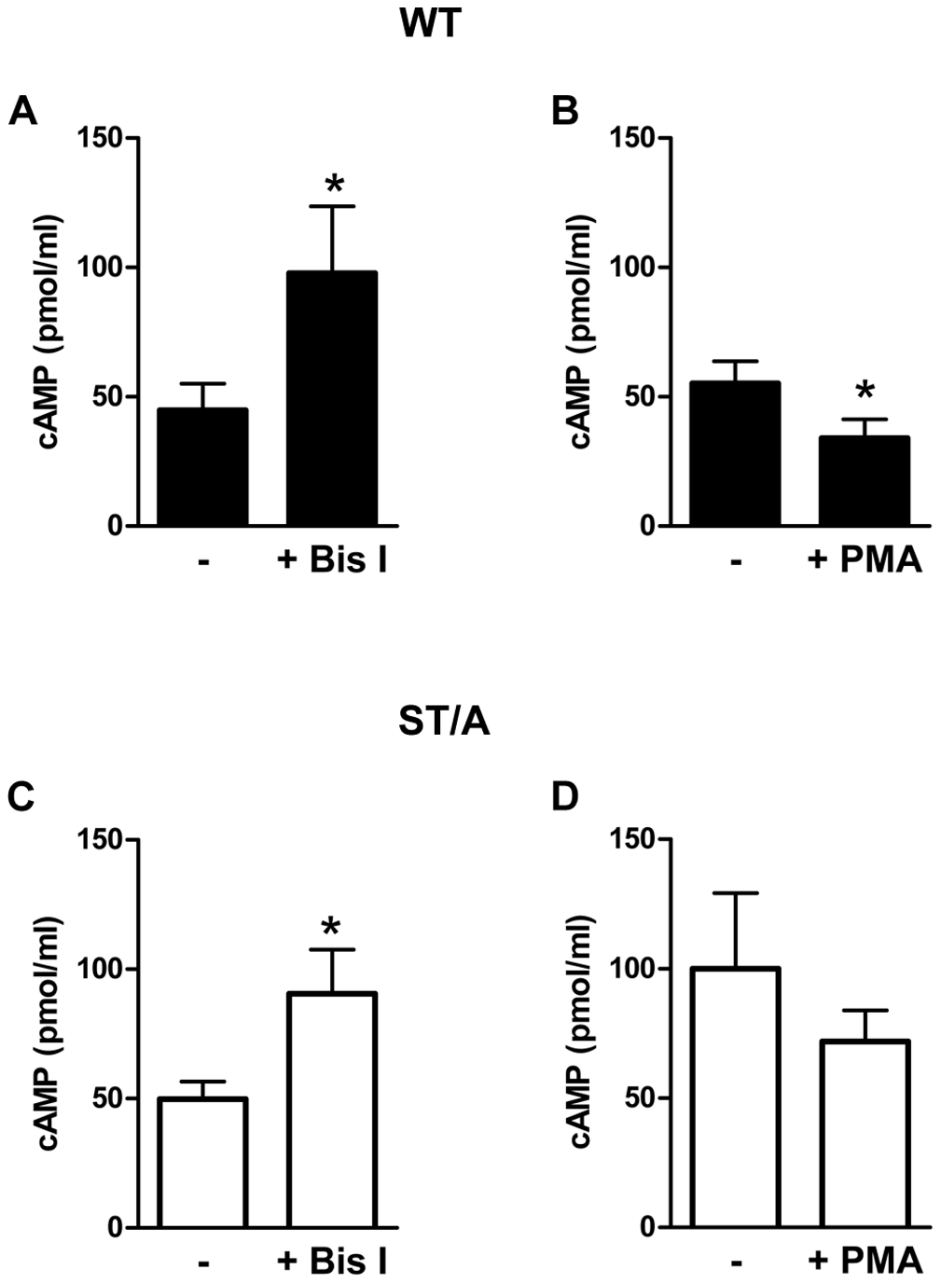
PKC inhibition increases intracellular cAMP and PKC activation decreases intracellular cAMP levels. **A-B).** HEK293 cells were transfected with GPR3-HA WT and 4 hr later treated with 5 µM Bis I (**A**) or 10 nM PMA (**B**). Following 18 to 24 hr treatment, cells were harvested for cAMP EIA. **C–D**) HEK293 cells were transfected with GPR3-HA ST/A and 4 hr later treated with 5 µM Bis I (**C**) or 10 nM PMA (**D**). Following 18 to 24 hr treatment, cells were harvested for cAMP EIA. Results are presented as mean ± S.E.M from 3–4 separate experiments. (*) indicates a significant difference in cAMP levels from “-” DMSO treated cells as determined by two-tailed paired Student’s *t* test (*p*<0.05). ∼170,000 cells lysed in 0.1 M HCl were used in the EIA assay, except for the GPR3-HA ST/A treated with Bis I and DMSO where ∼90,000 cells were used in order to keep the levels of cAMP on the standard curve.

As mentioned above, it is possible that other kinases are involved in regulating GPR3 activity. In addition to GRK, second messenger-dependent kinases including PKA and PKC can also phosphorylate GPCRs [65]. Signal termination in this manner involves a feedback loop in which second messengers produced by GPCR signaling activate PKA or PKC, which then phosphorylate the receptor and recruit β-arrestin. Unlike GRKs, these kinases do not discriminate between agonist-bound and agonist-free GPCRs [23]. PKA is not likely to be involved in regulating GPR3 because GPR3 does not contain a PKA consensus site; however, GPR3 has two predicted PKC sites within the third intracellular loop and the C-terminus [4], [66]. The threonine in the third intracellular loop is of particular interest because when mutated, intracellular cAMP levels increase more than 2-fold over WT (Data not shown). To test if GPR3-cAMP signaling can be modulated by PKC, we treated HEK293 cells transfected with GPR3-HA WT with a PKC inhibitor (Bis I) or a PKC activator (PMA). In response to Bis I treatment, cAMP levels significantly increased compared to DMSO-treated cells (Fig. 8A). In contrast, treatment with PMA significantly decreased cAMP levels compared to DMSO treatment, suggesting that PKC could regulate GPR3 signaling (Fig. 8B). Since the threonine in the third intracellular loop is a potential PKC site, it is possible that this mutant will not respond to Bis I or PMA treatment. However, we found that Bis I treatment increased cAMP levels and PMA decreased cAMP levels for the ST/A mutant (Fig. 8C and D). Together, these results demonstrate the potential for GPR3 regulation by PKC but the threonine residue in the third intracellular loop does not appear to be involved in this regulation. Further studies are needed to examine if other PKC isoforms, not targeted by Bis I or PMA, can regulate GPR3 activity. In addition, PKC is activated by G_q_-coupled receptors or by G_βγ_ and there is currently no evidence that GPR3 can signal through these pathways.

In summary, GPR3 is a constitutively active receptor that is an important regulator of meiosis in oocytes [7]–[9] and has a variety of functions in the brain [14]–[19]. In oocytes, GPR3 is localized in the plasma membrane and early endosomes and inhibiting endocytosis increases cAMP levels [31]. However, the mechanisms controlling GPR3 activity and subcellular localization, if any, have not yet been characterized. In the present study, we found that inhibition of endocytosis results in increased GPR3 at the cell surface and increased cAMP levels. Conversely, overexpression of GRK2 and β-arrestin-2 decreased both cell surface GPR3 and intracellular cAMP levels. Together, these results are consistent with the hypothesis that GPR3 signals at the cell surface and is susceptible to desensitization by a GRK2- and β-arrestin-2-dependent mechanism. The kinase activity of GRK2 is not required to diminish cAMP production by GPR3 but it is required to decrease cell surface GPR3 with β-arrestin-2. We also provide evidence that the serine or threonine residues in the third intracellular loop regulate GPR3 activity independently of GRK2 and PKC. Future studies are needed to determine how GRK2, PKC, and the serine and threonine residues in the third intracellular loop regulate GPR3 activity and if additional regulatory proteins interact with GPR3. It will also be interesting to examine if GPR3 is regulated by similar mechanisms in oocytes and if GPR3 localization and trafficking is perturbed in women with reproductive problems such as fertility or primary ovarian insufficiency. Several studies have examined whether mutations in GPR3 are present in women with primary ovarian insufficiency; however, no perturbations were found in the coding region of GPR3 in the populations included in these studies [67], [68]. Whether GPR3 localization or activity in the ovary is abnormally regulated in these women remains to be explored.
